# The effect of transcranial direct current stimulation of dorsolateral prefrontal cortex on performing a sequential dual task: a randomized experimental study

**DOI:** 10.1186/s41155-021-00195-8

**Published:** 2021-10-09

**Authors:** Rasool Abedanzadeh, Saeed Alboghebish, Parisa Barati

**Affiliations:** Department of Motor Behaviour, Faculty of Sport Sciences, Shahid Chamran University of Ahvaz, Ahvaz, Iran

**Keywords:** Selective attention, Reaction time, Processing information, Psychological refractory period, Stroop effect, tDCS

## Abstract

When it comes to simultaneous processing of two tasks, information processing capacity is usually below par and not desirable. Therefore, this preliminary study aimed to investigate the effect of transcranial direct-current stimulation (tDCS) of dorsolateral prefrontal cortex (DLPFC) on performing dual tasks. Twenty-six students (average age 25.2 ± 2.43 years) were selected and then randomly divided into experimental and sham groups. All of the participants conducted the Stroop effect test in a dual task situation before and after the tDCS. This test included two intervals between the stimuli of 100 and 900 ms. The results of mixed-ANOVA showed that the average second reaction time of the experimental stimulated group was reduced (in both dual tasks with congruent and incongruent stimuli) significantly after the tDCS. Therefore, it can be stated that the tDCS of the DLPFC increases the information processing speed and the capacity of attention and, as a result, decreases the effect of the psychological refractory period.

## Introduction

Attention is the capacity for processing the information we receive from the environment. This information processing capacity is limited for each individual. Each task requires part of this capacity; therefore, if the individual’s processing capacity needed for the simultaneous performance of two tasks is more than their total processing capacity, the efficiency of one or both of the ongoing tasks is reduced (Melzer, Kurz, Shahar, Levi, & Oddsson, 2007). In fact, when two tasks are performed simultaneously or with a systematic delay in dual task situations, the performance is disrupted (Pashler, 2000; Wickens & Kessel, 1980). In Welford’s single-channel theory (Welford, 1967), it is assumed that in the information processing channel there is a stage called the bottleneck, which prevents the simultaneous processing of two tasks. Therefore, as long as the first task has not passed through the information processing channel, the second task will not be processed. This delay causes the psychological refractory period (PRP), and the PRP is affected by stimulus-onset asynchrony (SOA). The PRP is decreased by increasing SOA, because in the long SOA there is more time to process S1 and when S2 is presented, S1 will be in a later stage of processing information (Fischer & Plessow, 2015; Strobach, Schütz, & Schubert, 2015). The short and long SOAs have different effects on the stages of processing information. Previous research has shown that the response selection stage is more affected by decreasing SOA since in short SOA, the bottleneck is occupied when the second stimulation arrives (Sommer, Leuthold, & Schubert, 2001).

Another important factor influencing attention and information processing is the Stroop effect. In the congruent condition of the Stroop effect, the identity of the word and the ink color is the same. Since reading the word is processed faster than saying the ink name, the response to stimulus is facilitated. For the incongruent condition, the ink color and the identity of the word are different. So, the ink color is selected for the response and the reading word is inhibited. The incongruent condition of the Stroop effect requires a high cognitive effort because it needs to identify the ink color and ignore the meaning of the word (Kapoula et al., 2010). For congruent and incongruent conditions of the Stroop task, reading the word may automatically activate a response that can facilitate the response selection stage or interfere with it (Jongen & Jonkman, 2008). Recently, there has been research demonstrating that Stroop interference could happen at the primary stages of information processing due to semantic and response interferences (Rezaei, 2019). In the standard Stroop task, first, the S1 is presented, and after 2 s, the S2 will be presented in other words there was a very large SOA between presenting stimuli (Fagot & Pashler, 1992). But, in the sequential dual task of Stroop task, the SOA between S1 and S2 is close together. For example, in some studies, it is set from 50 to 900 ms (Logan & Gordon, 2001). This effect is concerned with the performance defect of selective attention and inhibition. Inhibitory control plays a vital role in executive functions and regulates the thoughts and actions of individuals.

In behavioral problems, response inhibition deficits, which eventually lead to an inhibitory control deficit, are associated with developmental disorders such as attention deficit disorder. It is worth noting that inhibitory mechanisms are of different types: inhibition in situations where disturbing stimuli should be ignored and one stimulus is considered to be the target stimulus, the inhibition of a stimulus for which the individual performing a task has already received a reward, and inhibition in interference situations where certain aspects of the stimulus are aimed while other elements must be controlled (Dick et al., 2010). In the present study, this inhibition is assessed.

As to the areas involved in inhibitory control, the findings of neuroimaging studies have revealed that while inhibitory control is active, the dorsolateral prefrontal cortex (DLPFC) and anterior cingulate cortex are activated (Borst et al., 2014; Loftus, Yalcin, Baughman, Vanman, & Hagger, 2015; Yip, Lacadie, Sinha, Mayes, & Potenza, 2019). Studies relying on structural magnetic resonance imaging (MRI), functional magnetic resonance imaging (fMRI), and repetitive transcranial magnetic stimulation have provided strong evidence that defects in the left frontal area, especially in the prefrontal, are the underlying causes for impairing the inhibitory control (Rogasch, Daskalakis, & Fitzgerald, 2015; Vaidya et al., 1998). Although the neural and neuronal imaging studies show that the areas of the left prefrontal cortex play a role in inhibiting control and cognitive control, these areas are activated through the implementation of both *Go/no Go* Test and Stop Signal Task, and the DLPFC is active during the preparatory cue period for color naming in the Stroop test (MacDonald, Cohen, Stenger, & Carter, 2000; Van Holst, Van Holstein, Van Den Brink, Veltman, & Goudriaan, 2012). Executive functions facilitate goal-directed behaviors. Inhibitory control is a skill of executive functions that contribute to modulating the operation of various cognitive subprocesses that adjust the dynamics of human cognition (Strobach & Antonenko, 2017). The importance of executive functions on dual task is observed when two tasks are processed sequentially rather than simultaneously. In this situation, the limitation of capacity processing leads to response delaying. Further, in dual task situations, the response selection stage of central processing is correlated with executive functions (Pashler, 1994; Strobach et al., 2015).

Transcranial direct current stimulation (tDCS) is a neuromodulatory technique (Guleken, Eskikurt, & Karamürsel, 2020). A low direct-current is applied to the cortical regions, facilitating or inhibiting spontaneous neural activity (Brunoni et al., 2012). tDCS has two mechanisms affecting the performance: tDCS can induce the activation of voltage-gated pre- and postsynaptic sodium and calcium channels by subthreshold depolarization and also induce lasting cortical excitability elevations (Nitsche & Paulus, 2001).

tDCS of the brain has been extensively investigated in the last decade; it acts as a non-invasive, inexpensive, and safe option for cortical excitability by altering the resting potential of cortical neural cells. By connecting anodal (positive) and cathodal (negative) electrodes to the points of interest on the surface of the skull, this weak direct current leads to the stimulation of the lower neurons. Cathodal stimulation reduces cortical excitability, whereas anodal stimulation increases cortical excitability (DaSilva, Volz, Bikson, & Fregni, 2011). The permanence of excitability depends on the intensity and duration of receiving tDCS and has different effects on excitability. Receiving 5 and 7 min of tDCS decreases the motor cortical excitability for minutes after terminating the stimulation session. Receiving 9 min of tDCS leads to a higher permanence of excitability than receiving 1 h of tDCS. Also, it has been shown that receiving 5 min of tDCS leads to a higher permanence of excitability than receiving tDCS for 35 min (Nitsche et al., 2003; Nitsche & Paulus, 2001). The size of electrode is another important factor for excitability; tDCS to the DLPFC shorter than 25 cm^2^ is recommended for the best effect (Imburgio & Orr, 2018). Electrode positioning in transcranial electrical stimulation is crucial in determining the effectiveness of stimulation. To improve Stroop effect performance in the tDCS to the DLPFC, F3 and F4 (10-20 EEG system) as an anode electrode and extracranial cathodes are recommended (Imburgio & Orr, 2018). During stimulation, some slight side effects are observed, which include itching under the electrode and mild headaches, both during and after stimulation. These effects are seen in different brain regions in healthy subjects and patients with various neurological disorders (Utz, Dimova, Oppenländer, & Kerkhoff, 2010). Studies that have examined the effectiveness of tDCS in cognitive functions show the effects of inhibition and facilitation. For example, external DLPFC anodal stimulation improves the accuracy of performance in a word letter order test in healthy subjects, the N-back working memory test in patients with Parkinson’s disease, and the digit span test in patients with major depression after five sessions of stimulation (Utz et al., 2010). However, there is no agreement on the locus of bottleneck in the stages of dual task information processing. In the Stroop effect, the congruency is in association with the processes occurring at the stage of stimulus identification (Scerrati, Lugli, Nicoletti, & Umiltà, 2017), and it takes at least 100 ms of stimuli presenting to complete the occurring effect (SOA 100) (Dyer, 1974; Glaser & Glaser, 1982). Increasing SOA, decreasing second reaction time (RT2), and manipulating SOA are in association with the limitation of processing capacity (Fischer & Plessow, 2015). Recent research has shown that manipulating SOA will impact both first reaction time (RT1) and RT2 (Janczyk, Renas, & Durst, 2018). Therefore, the present study aimed to investigate the effect of tDCS to the DLPFC on the Stroop task in a sequential dual task setting, where participants responded to repeated presentations of Stroop word-color pairs with different SOAs. So, this study was conducted to respond to the following questions: does tDCS to the DLPFC affect the reaction time in short and long SOAs? Does tDCS to the DLPFC affect both S1 and S2 in a sequential dual task of the Stroop effect?

## Methods

### Participants

**Fig. 1:**
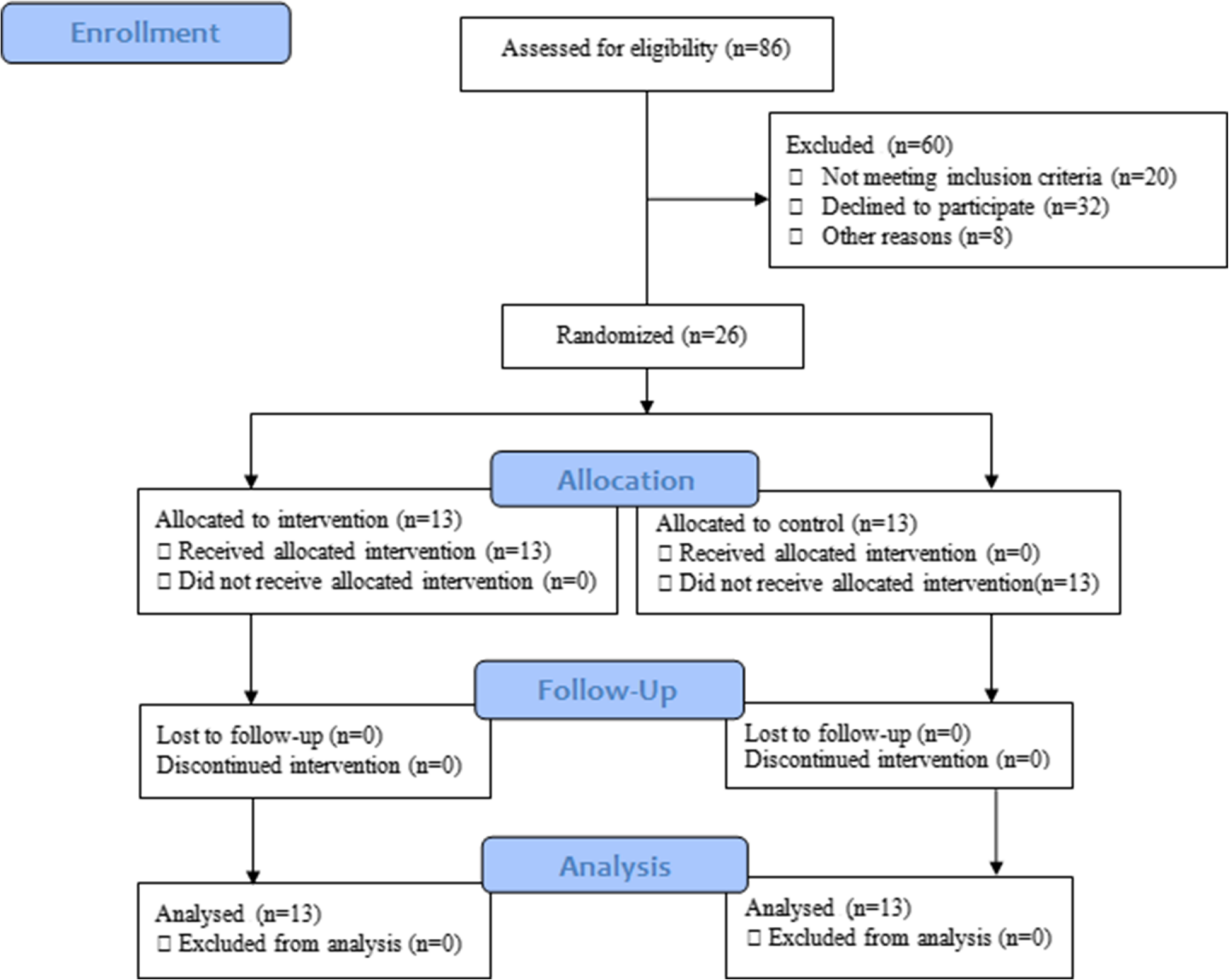
Flow diagram of participats

This study was an applied semi-experimental research with a pre-test post-test design. The statistical population included male and female students at the Shahid Chamran University of Ahwaz (Ahwaz, Iran) with an average age of 25.30 ± 2.43 years. To estimate the sample size, G*Power 3 was run with a confident interval of 0.05, statistical power of 0.95, and effect size of 0.25. Also, the selected statistical test was ANOVA: repeated measures, within-between interaction. The computed required sample size was 24. Therefore, with regard to falling probability, 26 of these students were selected by convenience sampling and divided into experimental and sham groups via simple randomization (Fig. 1). Each group included seven males and six females. Participants who had normal vision and no history of neurological disorders participated voluntarily in the study. Also, during the study, the participants were free to continue or discontinue the research process. Criteria for exclusion were having attention deficit disorders, mental disorders, or severe depression. Moreover, all participants had normal vision or corrected to normal and reported they had no history of neurological or mental disorders. Written informed consent from the participants was obtained. All participants had the right to withdraw from the research protocol at any time they wanted. This study has been approved by the ethics committee of the Shahid Chamran University of Ahvaz.

### Apparatus

#### tDCS

To stimulate the brain for this research, a transcranial direct-current stimulation system (Neurostim 2, Medina Teb Iranian Company) was used. The device has two completely separate channels, with each channel being independently adjusted to apply various types of stimuli. To stimulate the lateral-posterior prefrontal part, the Sponge-electrodes measuring 4 × 4 cm were used. The electrode size smaller than 25 cm^2^ was recommended to increase the executive function in tDCS to the DLPFC (Imburgio & Orr, 2018). According to 10-20 system, the anodal electrode between the F3 and C3 and the cathodal electrode on FP2 were placed. Also, a saline solution was used to wet the pads. When the connection of the electrode and scalp is disconnected, the apparatus will play a warning tone.

#### Stroop effect dual task device

A researcher-made device, whose software had been designed in Microsoft.NET framework, Microsoft Visual C++ (2015), was also employed to write the software. The device hardware includes an electronic circuit, an IC (Atmega32), a microcontroller, a number of micro switches, and a TTL serial converter. These components are mounted on the breadboard. The connection between the hardware and the computer is made via a USB port. The software of this device can adjust and select the type of sequence of the first and second stimuli aurally, neutrally, congruently, and incongruently. In this device, the interval between the two stimuli is adjustable, and the identity of the word and the ink color can be set for each trial and each stimulus of the dual task. The output data of this software, which includes the first and second reaction times, and the type of response selection, are reported in Microsoft Excel. The hardware of the device comprises a keyboard containing four blue, red, yellow, and green buttons (for responding to visual stimuli) and two triangular up and down buttons for aural stimuli. The keyboard is connected to a laptop with a 15.6 monitor screen via a USB port. The reliability of the device was estimated to be 0.82 through a pilot study carried out by the test-retest method on 20 participants from the Shahid Chamran University of Ahvaz. As for the validity, the concurrent validity of this device for measuring the reaction time was assessed. Pearson correlation coefficient between the reaction times in the tools was 0.80. It should be noted that, with regard to the speed of information transfer from the hardware to a laptop, the probability of time errors for the registered records was 0.001 s.

### Procedure

At first, the practice trials were conducted for the participants in two intervals (100 and 900 ms) between two stimuli in congruent and incongruent dual task conditions of the Stroop effect during the psychological refractory period. These SOAs were taken from similar studies (Fagot & Pashler, 1992; Logan & Gordon, 2001; Soutschek, Taylor, & Schubert, 2016). To remove the structural interference between the limbs, the two blue and green keys on the right side of the participants were to be responded with their right hand, while the red and yellow keys on their left side were to be responded with their left hand. In each trial of the dual task, both hands were used to return to the random stimuli, using the left and right side for S1 and S2. No trial was made to use one hand for two responses. The order of appearance of colors for all stimuli was random (randomization was done from a computer-generated sequence), and two different colors were used in each trial. To eliminate the participants’ sensitivity to color, all the possible sequences of color were presented. The words were BLUE, GREEN, RED, and YELLOW present randomly in green, blue, red, and yellow. The 16 possible dual task situations for congruent-congruent and 16 possible dual task situations for incongruent-incongruent were presented in each SOA.

After undergoing familiarization efforts, and while the participants were sitting on one chair in a 60-cm distance from the screen, they conducted the task trials. In two trials of each block (2 blocks) under the following conditions, they performed the Stroop effect dual task test. In the first block, at 100 ms SOA, the first and the second stimulus were provided at random in two congruent (matched ink color and word meaning, for example, the red word was written in red ink) or incongruent (unmatched ink color and word meaning, for example, the word blue was written in red ink) modes. The participants, regardless of the meaning of the word appearing on the screen, had to detect only the color, and by pressing the button (red, blue, yellow, or green) based on the color of the presented word, they had to respond as quickly as possible to the stimuli. The time interval between the two trials was considered 2 s, and the resting time between all blocks was 2 min (Logan & Gordon, 2001). There were two blocks, including SOA 100 ms block and SOA 900 ms block. In each block, 32 trials including 16 congruent-congruent dual tasks and 16 incongruent-incongruent dual tasks were presented. In the sequential dual task of Stroop task, the S1 was presented, and after the SOAs 100 or 900 ms, the S2 was presented.

The blocks and trials were given randomly; thus, the tests of congruent-congruent and incongruent-incongruent dual task were presented in random sequences in each SOA. The background color of the screen was white in all experiments. It is worth noting that the responses faster than 150 ms in the first stimulus and the responses longer than 2500 ms in the second stimulus were disregarded (Logan & Gordon, 2001). The pre-test and post-test conditions of the Stroop effect on the PRP were similar for both groups.

#### tDCS stimulation

**Fig. 2:**
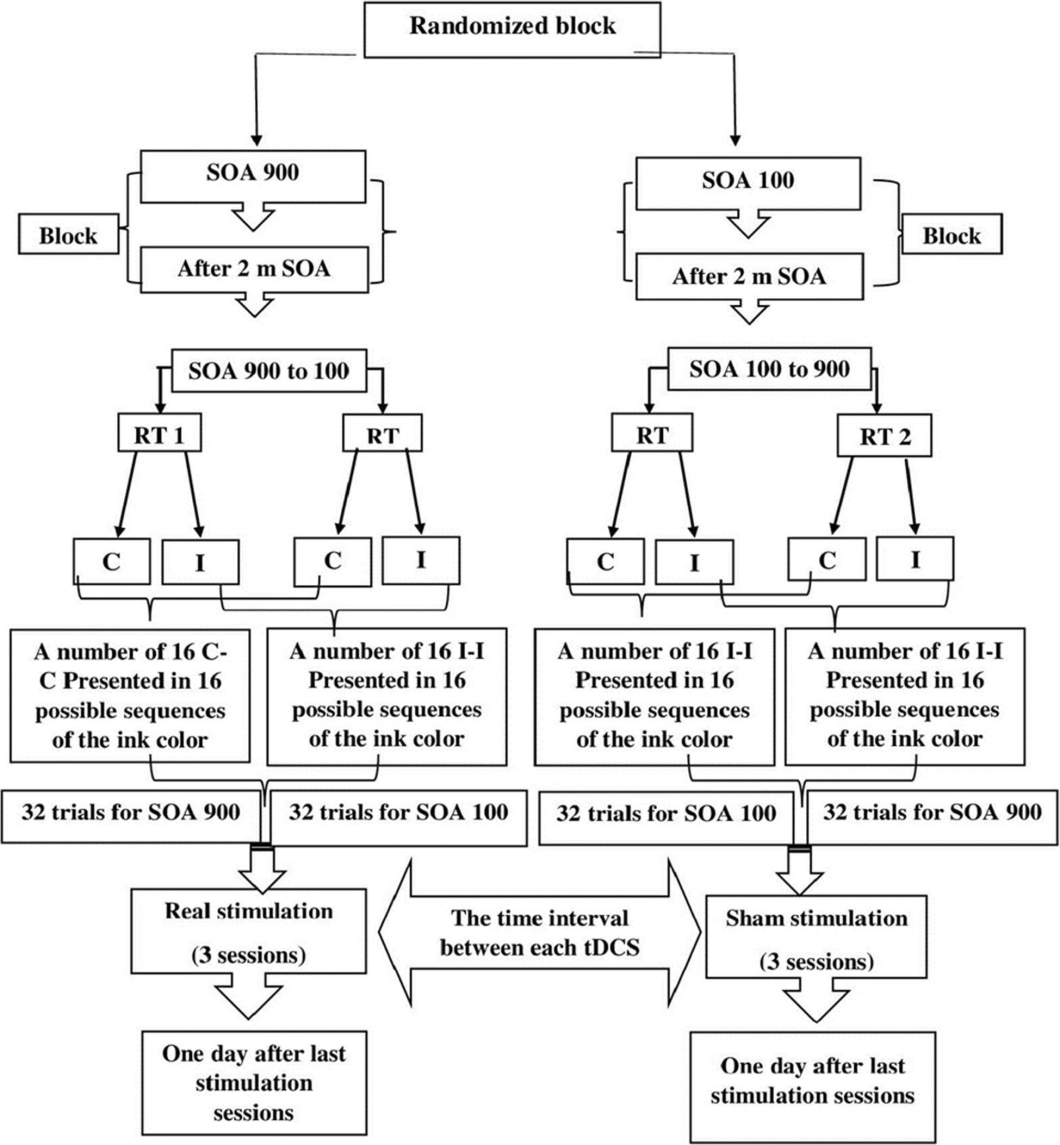
Schematic design of research. SOA, stimulus onset asynchrony; RT1, first reaction time; RT2, second reaction time; C, congruent; I, incongruent

The performances of the sham and experimental groups in each test of the Stroop effect were to be compared in dual task conditions before and after stimulation of tDCS. The participants underwent anodal stimulation (between F3 and C3 of the 10-20 system) of 1.5 mA for 20 min. The cathodal electrode was located at FP2 of the 10-20 system (Strobach & Antonenko, 2017). There was a 1-day interval between each tDCS session. After the pre-test, all participants were randomly divided into the experimental and sham groups. The electrodes of the tDCS device were connected to the same experimental sites in both groups, with the only difference in the sham group (Fig. 2). In the sham group, the electrical current of the electrodes was disconnected after the first 30 s of stimulation, and current intensity was gradually increased and decreased to diminish its perception (Boggio et al., 2008). The participants were blind to their group allocation. The data collection operator (Stroop software) did not know the type of tDCS. All the data collection steps were performed in the laboratory of the Shahid Chamran University of Ahvaz.

### Statistical analysis

Shapiro-Wilk’s test was implemented to check the normality distribution of the data. If data distribution was normal, then, to estimate the effect of tDCS on dual tasks, the two mixed-ANOVAs 2 (group) × 2 (SOA) × 2 (stimuli) × 2 (congruency) were performed on each phase (pre- and posttest) for the conditions, namely congruent-congruent and incongruent-incongruent, with the group as a between-subject factor and later three factors as within-subject factors. Then, to determine the performance differences between groups in each experimental condition, post hoc independent *t* tests were conducted separately for each condition of stimuli. The dependent variables were RT1 and RT2. All analyses were performed by SPSS version 23. The significance level was set at *p* < 0.05 for ANOVAs, and it was adjusted by Bonferroni correction for multiple comparisons which were conducted via independent t-tests. The data supporting the findings of this study are available from the corresponding author on reasonable request.

## Results

**Fig. 3:**
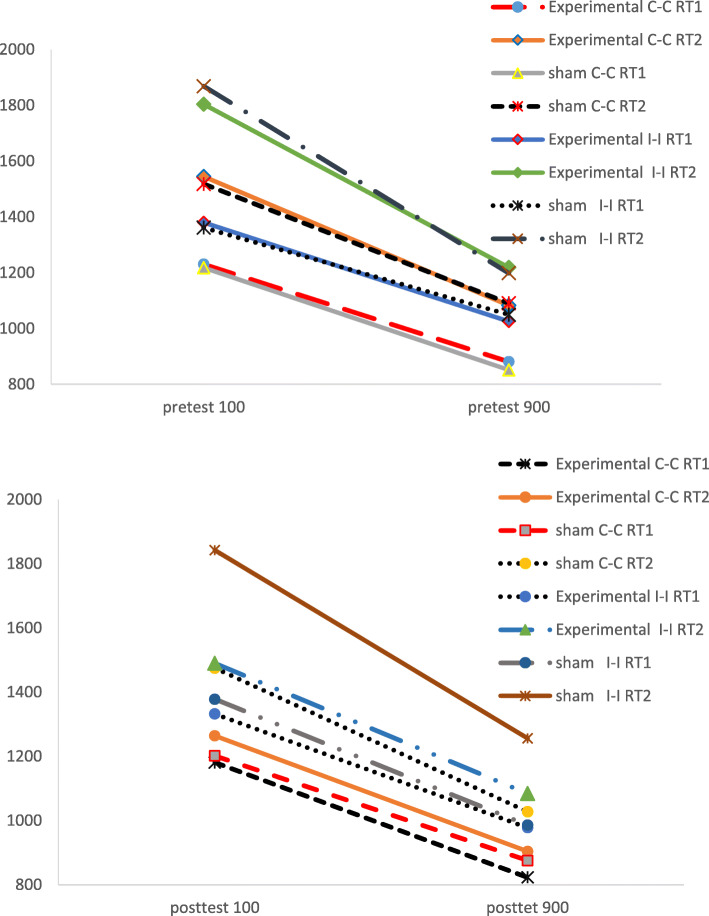
The means of RT1 and RT2 in the sham and experimental groups for each the pre-test (up) and post-test (down). C-C, congruent-congruent; I-I, incongruent-incongruent; RT1, first reaction time; RT2, second reaction time

The mean and the standard deviation of the reaction times to Stroop effect stimuli in dual stimulation conditions in pre-test and post-test are presented in Fig. 3.

To analyze the differences between the experimental and sham groups in the pre-test, mixed-ANOVA 2 (group) × 2 (SOA) × 2 (stimuli) × 2 (congruency) was run (Table 1). The results did not reveal any significant effect of the group (*F*_(1, 24)_ = 141.58, *p* = .953, *η*_p_^2^ = .0001) and the interactions including group factor (*p* > .05). Therefore, it was specified that there was no significant difference between the experimental and sham groups in the pre-test stage.

**Table 1:** The result of mixed-ANOVA tests between the sham and experimental groups in the pre-test

Source	*dfN*	*dfD*	*F*	*Sig.*	_*Ƞ*p_^2^
Stimulus	1	24	141.58	.0001	.85
Stimulus × group	1	24	0.787	.38	.03
SOA	1	24	590.91	.0001	.96
SOA × group	1	24	0.027	.87	.001
Congruency	1	24	456.64	.0001	.95
Congruency × group	1	24	0.315	.58	.01
Stimulus × SOA	1	24	8.40	.008	.26
Stimulus × SOA × group	1	24	0.294	.59	.01
Stimulus × congruency	1	24	3.16	.09	.12
Stimulus × congruency× group	1	24	0.020	.89	.001
SOA × congruency	1	24	52.89	.0001	.69
SOA × congruency× group	1	24	0.538	.47	.02
Stimulus × SOA × congruency	1	24	21.60	.0001	.47
Stimulus × SOA × congruency× group	1	24	4.004	.06	.14
Group	1	24	0.004	.95	.0001

dfN, numerator degrees of freedom; dfD, denominator degrees of freedom; SOA, stimulus onset asynchrony

To examine the differences between the groups in the post-test, the mixed-ANOVA 2 (groups) × 2 (SOA) × 2 (stimuli) × 2 (congruency) was run (Table 2). As expected, results showed there were a significant main effect on the group (*F*_(1, 24)_ = 31.62, *p* = .0001, *η*_p_^2^ = .56) and significant interactions between stimulus × congruency × group (*F*_(1, 24)_ = 5.34, *p* = .02, *η*_p_^2^ = .18) and between SOA× congruency× group (*F*_(1, 24)_ = 14.64, *p* = .001, *η*_p_^2^ = .37).

**Table 2:** The result of mixed-ANOVA tests between the sham and experimental groups in the post-test

Source	*dfN*	*dfD*	*F*	*Sig.*	_*Ƞ*p_^2^
Stimulus	1	24	269.44	.0001	.92
Stimulus × group	1	24	3.07	.09	.11
SOA	1	24	623.33	.0001	.96
SOA× Group	1	24	4.51	.04	.16
Congruency	1	24	254.12	.0001	.91
Congruency × group	1	24	53.95	.0001	.69
Stimulus × SOA	1	24	5.216	.03	.18
Stimulus × SOA× group	1	24	2.26	.15	.09
Stimulus × congruency	1	24	20.40	.0001	.46
Stimulus × congruency × group	1	24	5.37	.03	.18
SOA× congruency	1	24	29.11	.0001	.55
SOA× congruency × group	1	24	14.64	.001	.38
Stimulus × SOA× congruency	1	24	2.1	.16	.08
Stimulus × SOA× congruency × group	1	24	0.08	.78	.003
Group	1	24	31.16	.0001	.56

dfN, numerator degrees of freedom; dfD, denominator degrees of freedom; SOA, stimulus onset asynchrony

According to the significant three-way interaction in Table 3, for further investigation of significant interaction, multiple comparisons via independent *t* tests adjusted by Bonferroni correction were conducted (Table 3).

**Table 3:** The results of independent *t* tests between sham and experimental groups

		Congruent-congruent			Incongruent-incongruent		
Stimulus	SOA	*t*	*df*	*Sig.*	*t*	*df*	*Sig.*
S1	100	− 0.6	24	.55	− 1.1	24	.28
S2	100	− 4.82	24	.0001	− 7.83	24	.0001
S1	900	− 1.49	24	.15	− 0.21	24	.84
S2	900	− 3.63	24	.001	− 4.29	24	.0001

S1, stimulus 1; S2, stimulus 2; SOA, stimulus onset asynchrony

With regard to the results in Table 3, post-hoc independent *t* tests were conducted. There was a significant difference between the experimental group and the sham group regarding the time of the second reaction in the independent *t* test at the interval of 100 ms in the congruent-congruent (*t*_(24)_ = − 4.82, *p* = .0001) and the incongruent-incongruent task (*t*_(24)_ = − 7.32, *p* = .0001) and at the interval of 900 ms in the congruent-congruent (*t*_(24)_ = − 3.62, *p* = .001) and the incongruent-incongruent task (*t*_(24)_ = − 4.29, *p* = .0001). According to this result, the tDCS to the DLPFC method has a significant effect on the dual task in SOA, inhibitory control, and attention.

## Discussion

This study aimed to investigate the effect of tDCS of the prefrontal region of the cortex on the psychological refractory period. There was a significant difference between the experimental and the sham groups in terms of the congruent-congruent or incongruent-incongruent dual tasks on the psychological refractory period. In the post-test phase, at two intervals between the two stimuli (100 and 900 ms), it seems that the tDCS of the DLPFC leads to a reduction in the reaction time, given the fact that doing dual tasks increases the activity in the DLPFC (Strobach & Antonenko, 2017). This increased activity has been observed in dual task studies using fMRI (Logothetis, 2008). Therefore, the electrical stimulation of this area seems to reduce the processing time of information. Hsu et al. (2015) achieved similar results (Hsu, Zanto, Anguera, Lin, & Gazzaley, 2015). This finding is supported by the fact tDCS modulates neurotransmitter concentration (Nitsche et al., 2004). This effect modulates cognitive functions. The last finding suggested tDCS also has an effect on deeper brain regions, for example, tDCS to the DLPFC modulates dopamine-GABA functions in the basal ganglia-cortical circuit (Bunai et al., 2021).

The main effect of SOA is significant. Manipulating SOA has a different effect on RT of dual task. tDCS to the DLPFC led to faster response in the experimental group in both short and long SOAs. In the short SOA, when the second stimulus is received, the first stimulus occupies the information processing capacity so that there will be a delay in processing the second stimulus. This delay in the short SOA is more than in the long SOA (Fischer & Plessow, 2015; Strobach et al., 2015). Also, the numerical value of the *t* test showed that both the congruent-congruent and I congruent-I congruent dual task in the short SOA have a higher value of significance. Perhaps the tDCS to the DLPFC increases the capacity of attention. Attention capacity is an essential element in processing information. In the short SOA, if two stimuli are processed simultaneously, the interference may start in the early stages of information processing and continue to the later stages. However, if the two stimuli are processed serially, the interference may occur in the same stages of information processing. In the long SOA, the interference may occur in two different stages (for example, the second stimulus is at the response selection stage and the first stimulus is at the performance stage). These differences led to a small effect size of the interaction between the group and SOA.

Improving the response time of the S2, or reducing the effect of the psychological refractory period in the experimental group, indicates the positive impact of electrical stimulation of the prefrontal part of the cortex on the speed of information processing. Reviewing most of the psychological refractory period studies shows that information processing capacity is limited in humans (Fischer & Plessow, 2015; Strobach et al., 2015). When processing two tasks during the bottleneck stage, the simultaneous processing of two tasks is avoided. During one of the three stages of information processing, including stimulus identification, response selection, and response programming, the bottleneck prevents simultaneous processing of two tasks. Therefore, the second task should wait until the first task passes through the bottleneck. It seems that tDCS over the DLPFC modulates the speed or the capacity of information processing. This finding cannot be used to assume that the passing bottleneck has no delaying. However, it show shows the effect of tDCS on the bottleneck. Still, it increases the speed of the passage or processing of the first stimulus. Therefore, the effect of the psychological refractory period is decreased (Fischer & Plessow, 2015; Strobach et al., 2015).

tDCS of the DLPFC, as a neurotherapeutic technique, applies a direct and weak current to different cortical regions, depending on the purpose of the treatment or research. Thus, it physiologically inhibits or facilitates the activity related to the desired movement. Following the application of tDCS and cortical excitability modification, the motor-evoked potentials are facilitated in the area under the anode electrode, and cortical plasticity associated with the improvement of motor execution occurs (Stagg & Nitsche, 2011).

This study showed that tDCS of the DLPFC reduces the reaction time to Stroop effect stimuli in both congruent and incongruent situations. Therefore, the DLPFC region is involved in the processing of Strop effect stimuli. This role may be in the naming process or the speed of information processing of the Stroop effect stimuli. Also, tDCS improves performance by affecting the DLPFC area. An increase in processing speed has also been observed in the study of Marvis et al. (Mervis, Capizzi, Boroda, & MacDonald III, 2017).

Due to the decrease in the reaction time of the first stimulus, there will be more time available for the naming process of the second stimulus. Therefore, a decrease during the reaction time of both stimuli is quite visible. However, after responding to the first stimulus and completing the apparent process of returning by pressing a button on the keyboard, the processing of the stimulus will continue. So, after answering, the correct answer to the stimulus will be comprehended. It seems that the improvement has been achieved. In addition to the reaction time, it has also affected the post-response processing. Therefore, even in long SOAs, the decrease in the reaction time of the second stimulus can be observed. The results of this study are consistent with the decrease of reaction time with DLPFC electrical stimulation as reported in the study of Dedoncker et al. (2016) (Dedoncker, Brunoni, Baeken, & Vanderhasselt, 2016).

Since the anodal tDCS on the DLPFC improved the cognitive dual task costs, and the DLPFC has a role in naming the color of the Stroop effect, improving the reaction time of the Stroop effect is justifiable. Seemingly, there are additional roles of DLPFC in the Stroop effect in dual task conditions. In the incongruent condition identifying the color of the word ink, ignoring the meaning is more complicated than congruent condition. Improvement of inhibitory control is observed in incongruent Stroop effect conditions. This finding shows the importance of DLPFC in inhibitory control. DLPFC area has a relation with selective attention (Stroop effect), especially on color naming of incongruent-incongruent dual task. tDCS of DLPFC has a considerable impact on congruent-congruent dual task; this improvement is observed in both SOAs. Therefore, the tDCS can modify the naming of the color in both conditions of the Stroop effect. According to the central capacity sharing model (Tombu & Jolicœur, 2003), when two or more tasks are to be done in a short time, implementing tasks simultaneously is prone to interference (Fischer & Plessow, 2015; Strobach et al., 2015). Contrary to the single-channel theory or the bottleneck model, various cognitive operations are planned by limited resources in this model. This model is based on the fact that two stimuli and two responses are performed in parallel and simultaneously. The limited sources are shared between the two processes, and no unique processing occurs at the processing stage (Fischer & Plessow, 2015; Strobach et al., 2015). It seems that the transcranial electric stimulation increases the capacity of information processing, which increases the simultaneous processing of two tasks. However, given that the first stimulus occupies more capacity of attention resources, the effect of the refractory period is still visible, though this effect is significantly reduced. The two limitations of this study were the small sample size and the lack of access to fMRI and EEG equipment. Considering the limitations, it is suggested that future studies evaluate brain waves and the structural and functional brain before and after performing tDCS on DLPFC.

## Conclusion

tDCS of DLPFC is a method which aims to improve the speed of information processing. Also, given that the tDCS of DLPFC increases the capacity of information processing, it is recommended that this method be used to improve the cognitive ability of individuals. It seems that the DLPFC has an important role in processing the Stroop effect in dual task situations.

## Data Availability

The datasets used and/or analyzed during the current study are available from the corresponding author on reasonable request.
